# Prevalence and Patterns of Multimorbidity among Elderly People in Rural Bangladesh: A Cross-sectional Study

**DOI:** 10.3329/jhpn.v29i4.8458

**Published:** 2011-08

**Authors:** Masuma Akter Khanam, Peter Kim Streatfield, Zarina Nahar Kabir, Chengxuan Qiu, Christel Cornelius, Åke Wahlin

**Affiliations:** ^1^icddr,b, GPO Box 128, Dhaka 1000, Bangladesh; ^2^Ageing Research Centre, Department of Neurobiology; ^3^Division of Nursing, Care Sciences and Society, Karolinska Institutet, Stockholm, Sweden,; ^4^Department of Psychology, Stockholm University, Stockholm, Sweden

**Keywords:** Cross-sectional studies, Elderly, Morbidity, Multimorbidity, Bangladesh

## Abstract

Data on multimorbidity among the elderly people in Bangladesh are lacking. This paper reports the prevalence and distribution patterns of multimorbidity among the elderly people in rural Bangladesh. This cross-sectional study was conducted among persons aged ≥60 years in Matlab, Bangladesh. Information on their demographics and literacy was collected through interview in the home. Information about their assets was obtained from a surveillance database. Physicians conducted clinical examinations at a local health centre. Two physicians diagnosed medical conditions, and two senior geriatricians then evaluated the same separately. Multimorbidity was defined as suffering from two or more of nine chronic medical conditions, such as arthritis, stroke, obesity, signs of thyroid hypofunction, obstructive pulmonary symptoms, symptoms of heart failure, impaired vision, hearing impairment, and high blood pressure. The overall prevalence of multimorbidity among the study population was 53.8%, and it was significantly higher among women, illiterates, persons who were single, and persons in the non-poorest quintile. In multivariable logistic regression analyses, female sex and belonging to the non-poorest quintile were independently associated with an increased odds ratio of multimorbidity. The results suggest that the prevalence of multimorbidity is high among the elderly people in rural Bangladesh. Women and the non-poorest group of the elderly people are more likely than men and the poorest people to be affected by multimorbidity. The study sheds new light on the need of primary care for the elderly people with multimorbidity in rural Bangladesh.

## INTRODUCTION

It is projected that, by 2020, there will be one billion elderly persons (≥65 years) in the world, 71% of whom will live in low-income countries (1). The number of elderly persons in Bangladesh was projected to double from 7.8 million in 2001 to 16.2 million by 2025 (2). The country is currently undergoing both epidemiologic and demographic transitions, where the decline in both fertility and mortality rates in early life have resulted in increased life expectancy. According to the estimation of the United Nations, life expectancy at birth is expected to increase to 74 years in 2025 (3). Based on data from Bangladesh, life expectancy at birth is expected to be 76.9 years for men and 85.1 years for women in 2015, calculated based on the scientific report of icddr,b (4). At present, based on icddr,b data, life expectancy at 60 years is additional 17.6 years for men and 18.9 years for women (4).

The fact that more and more people are reaching their older adulthood has resulted in a change in the disease pattern such that chronic medical conditions have become prominent also in low-income populations. Chronic health conditions are now common in elderly persons, and the prevalence of multiple chronic conditions is expected to increase (5). Chronic diseases, by nature, will accumulate with ageing and present as multiple morbidities.

Multimorbidity is defined as simultaneous occurrence of several adverse medical conditions in the same person (6). The prevalence of multimorbidities has often been investigated, for example, in Europe (7), Australia (8), and the United States (9). Numerous studies have examined the distribution of multimorbidity among older persons in developed nations (7-9) but available literature on multimorbidity among the elderly people in developing countries is limited. In India, Joshi reported in 2003 that 83% of the elderly people had more than three morbidities (10). Purty, in 2006, reported that the average number of morbidities per person was 2.77 among the elderly people of rural India (11). In China, 21.7% of rural elderly people have at least two morbidities, and 15.9% have three or more morbidities (12). Little is known about the prevalence of morbidity among elderly persons in rural Bangladesh. Also, there is scanty information on the distribution of chronic conditions and multimorbidity by socioeconomic status.

Studies have adopted various ways of defining multimorbidity, which has yielded prevalence rates that vary according to the definition. Measures used for exploring co-occurrence of multiple diseases include Charlson Co-morbidity Index (13), the Index of Co-existent Disease (ICED) (14), etc. These indices usually do not cover the overall conditions affecting the population and often require medical records or skilled clinicians. Instead, data are often obtained from various surveys (9), administrative databases (5), or computerized networks of family practices (7). Other commonly-used methods include personal interviews, self-reports, and clinical examinations (15).

One approach for quantitative evaluation of the health status of elderly persons is to count the number of medical conditions (multimorbidity) they are affected by. A chief advantage of this procedure is simplicity, especially when a large number of diseases are evaluated. Obviously, the prevalence of multimorbidity largely depends on the number of diseases included for study, and the diagnostic criteria used. Generally, the higher the number of diseases studied, the higher the occurrence of multimorbidity. Using the same dataset, a study found that when all chronic conditions reported up to the date of interview are included, 63% of participants had two or more conditions compared to 49% of participants in an alternative analysis that considered only the eight most common conditions (9).

As with health and diseases, multimorbidity also requires attention by socioeconomic status. Health inequality by socioeconomic status is present in both high-income and low-income countries (16,17). People with low socioeconomic status suffer from more diseases than those with higher socioeconomic status throughout their lifespan. As newborns, they suffer from health problems, such as premature birth, low birthweight and, later on, from chronic diseases, such as diabetes or heart disease. They also suffer from infectious diseases or disabilities, such as blindness (16). In short, the poor people suffer from more ill-health and die at a younger age compared to their better-off counterparts (18). Hence, in a lifespan perspective, this may result in higher morbidity-prevalence figures among economically better-off persons since they tend to survive into old age.

Having multiple chronic medical conditions is associated with poor outcomes, such as poor quality of life (19), longer hospital stays, more post-operative complications, a higher cost of care, and higher mortality. Multimorbidity also affects care and may result in complex self-care needs (20), challenging organizational problems (accessibility, coor­dination, consultation time), polypharmacy, increased use of emergency facilities, difficulty in applying guidelines, and fragmented, costly, and ineffective care (21).

The main purpose of this study was to determine the prevalence and pattern of multimorbidity in terms of distribution by demographics and socioeconomic status among the elderly people in rural Bangladesh.

## MATERIALS AND METHODS

### Participants

Study participants were part of the study ‘Poverty and Health in Ageing’, a collaborative project between the Aging Research Centre at Karolinska Institutet (KI), Sweden, and icddr,b. This cross-sectional study was conducted among persons aged 60 years or older in rural Matlab in Bangladesh. Since 1966, icddr,b has been maintaining a surveillance system, currently known as Health and Demographic Surveillance System (HDSS), in this area. The HDSS covers a population of approximately 220,000 across 142 villages where regular update of all vital events is maintained. For administrative purposes, the total Matlab surveillance area is divided into seven blocks. For this study, we selected two blocks which are near the main Matlab Hospital.

Of 850 elderly individuals randomly selected from the two blocks, 63 died before data-collection, 38 refused to participate, 11 migrated, 93 could not be reached, 18 were registered twice in the surveillance database, and two were aged less than 60 years. Thus, 625 persons were interviewed in their homes, of whom 473 (75.7%) participated in clinical examinations. Complete information for the current study was available for 452 persons.

### Data-collection

Data were collected during July 2003–March 2004. Initial contact was made with individuals and their families to provide information to them about the study and to obtain informed consent. Data were then collected for all the participants during two sessions on two separate days. Data relating to their demographics and socioeconomic status were collected during interview in the home. Socioeconomic status was measured by literacy and asset index. Literacy was defined as the ability to read and write Bangla.

Information on asset index was separately collected from the surveillance database of the HDSS of icddr,b. The asset index is based on household assets and housing characteristics, such as bed, mattress, quilt, cooking-pots, watch, chair, clothing cabinet, radio, television, bicycle, boat, cows, and electricity. Using a variable reduction technique, these assets and characteristics were combined into a single variable. After ranking this variable from low to high, households were divided into five equal-sized groups—the poverty quintiles. Details on calculation of the asset index can be found in other publications from the Matlab HDSS (17). The poorest group of people was defined as in the lowest quintile of their assets.

Clinical examinations were performed at the local health centre of icddr,b by physicians, and peripheral blood samples were taken for further laboratory analyses. Diagnoses of diseases were done and recorded by two physicians and then evaluated separately by two senior physicians/geriatricians.

### Definition of multimorbidity

Multimorbidity was defined as suffering from two or more of the chronic medical conditions. The medical conditions used for defining multimorbidity in this study were the following:

*Arthritis:* Diagnosis was based on the reporting of (a) previous diagnosis by a doctor and having painful or stiff joints during the current clinical examination, or (b) ever having swollen joints.

*Stroke:* Diagnosis was based on physical examinations: presence of hemi or mono paresis judged to be of central origin or presence of pseudobulbar symptoms (dysarthria, dysphasia).

*Obesity:* It was defined as body mass index (BMI) of >27.5 kg/m^2^, the World Health Organization (WHO) cut-off for the Asian populations (22).

*Signs of thyroid hypofunction:* This condition was diagnosed from history as presence of any swelling in the neck and/or if thyroid stimulating hormone level was more than 5 μIU/mL.

*Obstructive pulmonary symptoms*: This condition was diagnosed from clinical examination: presence of abnormal breathing sound, ronchi, crepitation, or wheeze in the left or right lung.

*Symptoms of heart failure:* This condition was diagnosed by physical examinations: engorged neck-vein and presence of abnormal breathing sound (crepitation) in the left or right lung, and from history of difficulties in breathing or shortness of breath in minor exertion or presence of peripheral cyanosis or peripheral oedema.

*Impaired vision:* This condition was diagnosed by measuring visual acuity with a Snellen's chart, if the measurement was other than 6/6.

*Hearing impairment:* It was diagnosed by examining the ear with a tuning fork. A tuning fork was stroked and held in front of the ear (AC), then again stroked and held over the mastoid process, the bone behind the ear (BC). The sound produced by the vibrating tuning fork was compared: bone conduction (BC) and air conduction (AC). If BC < AC or AC < BC (23).

*High blood pressure:* It was defined as having systolic blood pressure (BP) of ≥140 mm Hg or diastolic BP of ≥90 mmHg (23).

### Analyses of data

Data are presented with mean [standard deviation (SD)] for continuous variables and with proportion for categorical variables. The overall and gender-specific prevalence rates of multimorbidity were calculated. The sample was divided into two age-groups (60-69 years and 70+ years). The cate-gorical variables were compared by chi-square statistics. Logistic regression analyses were performed to estimate odds ratios (ORs) and 95% confidence intervals (CIs) of multimorbidity associated with various factors, with and without adjustment for other explanatory variables. Data were analyzed using the SPSS software (version 11.5) for Windows.

### Ethical approval

Ethical permissions for the project were obtained from both Bangladesh (icddr,b) and Sweden (KI).

## RESULTS

The mean age of the participants was 69.5 (SD 6.8) years, the range being 60-92 years. Fifty-five percent of the participants were women, 60% were illiterate, and 17.5% belonged to the poorest quintile. The majority (53.7%) had multiple medical conditions (Fig.), and more than 84% had at least one condition. On average, women had more medical conditions than men (1.87 vs 1.27, p<0.0001).

**Fig.**
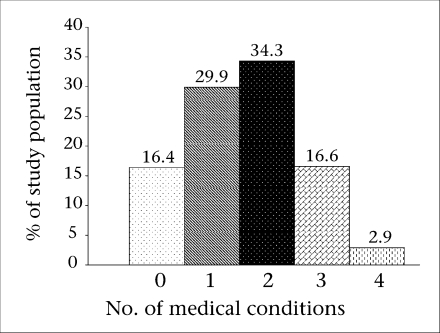
Distribution of medical conditions in study population by the number of conditions

The prevalence of multimorbidity was significantly higher among women than among men, among illiterate than among literate persons, among persons who belonged to the non-poorest quintiles than among the poorest quintile, and also significantly higher among single than among married persons (Table 1).

**Table 1.** Characteristics of study population (n=452) by multimorbidity*

			Multimorbidity				
Characteristics	Participants		Yes (n=243)		No (n=209)		p value
	No.	%	No.	%	No.	%	
Age-group (years)								
60-69	270	59.7	137	50.7	133	49.3	
70+	182	40.3	106	58.2	76	41.8	0.070
Sex							
Men	204	45.1	81	39.7	123	60.3	
Women	248	54.9	162	65.3	86	34.7	<0.0001
Literacy							
Illiterate	291	64.4	158	58.7	111	41.3	
Literate	161	35.6	83	46.1	97	53.9	0.006
Asset index							
Poorest	79	17.5	34	43.0	45	57.0	
Non-poorest	349	82.5	195	55.9	154	44.1	0.026
Marital status							
Single	189	42.1	119	63.0	70	37.0	
Married	260	57.9	122	46.9	138	53.1	0.001

*Multimorbidity was defined as having at least two of nine chronic medical conditions

**Table 2.** Frequency (prevalence per 100 people) of 9 individual chronic health conditions used in defining multimorbidity by sex (n=452)

Chronic health condition	Total population		Men (n=204)		Women (n=248)		p value*
	No.	%	No.	%	No.	%	
Arthritis	260	57.5	113	55.4	147	59.3	0.231
Hypertension	175	38.7	74	36.3	101	40.7	0.192
Impaired vision	161	35.6	8	3.9	153	61.7	<0.0001
Signs of thyroid hypofunction	48	10.6	18	8.8	30	12.1	0.166
Obstructive pulmonary symptoms	31	6.9	27	13.2	4	1.6	<0.0001
Symptoms of heart failure	21	4.6	13	6.4	8	3.2	0.088
Hearing impairment	11	2.4	2	1.0	9	3.6	0.062
Obesity	11	2.4	3	1.5	8	3.2	0.186
Stroke	4	0.9	1	0.5	3	1.2	0.388

*p value is for the test of difference between men and women

**Table 3.** Frequency (prevalence per 100 people) of chronic medical conditions by age group and sex

	Age							
No. of chronic conditions	60-69 years (n=270)				70 years and above (n=182)			
	Men (n=123)		Women (n=147)		Men (n=81)		Women (n=101)	
	No.	%	No.	%	No.	%	No.	%
No disease	32	26.0	14	9.5	36	44.4	70	69.3
1 disease	16	13.0	36	24.5	5	6.2	31	30.7
2 diseases	29	23.6	56	38.1	31	38.3	39	38.6
3 or more diseases	16	13.0	36	24.5	5	6.2	31	30.7

Table 2 shows the prevalence of chronic health conditions used for defining multimorbidity in the study. The most common disorders were arthritis (57.5%) and hypertension (38.7%). The prevalence of arthritis was significantly higher among the illiterate than among the literate group (62.1% vs 50.0%, p=0.007) and also higher among persons belonging to the non-poorest group than among persons in the poorest quintile (59.5% vs 48.1%, p=0.041) (data not shown).

Hypertension was significantly more common in the older age-group than in the younger group (45.0% vs 32.9%, p=0.006). It was also slightly more frequent among persons belonging to the non-poorest quintile than among persons in the poorest quintile (40.2% vs 31.6%, p=0.097) (data not shown).

Table 3 shows the prevalence of multimorbidity by age-groups (60-69 years and ≥70 years) and sex according to the number of adverse medical conditions. The rates of prevalence of multimorbidi-ty were significantly higher among women than among men in both the age-groups.

In a series of multivariate logistic regression analyses, gender and asset index were independently associated with multimorbidity (Table 4). Literacy and marital status had a crude association with multimorbidity, which disappeared after adjusting for other sociodemographic variables.

## DISCUSSION

We found that the overall prevalence of multimorbidity was 53.8% among the elderly people and that there were higher rates of prevalence among women, illiterates, and persons who were single or who belonged to the non-poorest quintiles. Overall, this study has shown a high prevalence of multimorbidity among the elderly people in rural Bangladesh, similar to what is frequently reported from many developed nations, e.g. Europe (61-80% in the Dutch population, 55% in the Swedish population) (6,24), Australia (75%) (8), and North America (65%) (9), although the criteria or definitions were not identical in those studies.

**Table 4.** Unadjusted and adjusted odds ratios and 95% confidence intervals of multimorbidity: results from logistic regression analyses

		Multimorbidity	
Characteristics	%	Unadjusted OR (95% CI)	Adjusted OR (95% CI)‡
Age-group (years)		1.00	1.00
60-69	50.7	1.35 (0.93-1.98)	1.29 (0.84-1.97)
70+	58.2		
Gender			
Men	39.7	1.00	1.00
Women	65.3	2.86 (1.95-4.20)	3.32 (1.88-5.86)
Literacy*			
Illiterate	58.7	1.00	1.00
Literate	46.1	0.60 (0.41-0.88)	0.85 (0.54-1.32)
Asset index†			
Poorest	43.0	1.00	1.00
Non-poorest	55.9	1.68(1.02-2.74)	1.93(1.14-3.27)
Marital Status*			
Single	63.0	1.00	1.00
Married	46.9	0.52 (0.36-0.76)	1.20 (0.68-2.11)

*Data on literacy and marital status were missing for 3 participants;

† Data on asset index were missing for 24 persons;

‡ ORs (95% CIs) were derived from the model that included age, gender, literacy, asset quintiles, and marital status;

CI=Confidence interval;

OR=Odds ratio

Female sex was associated with a higher prevalence of multimorbidity among this population. In both the age-groups, women had significantly higher proportions of multimorbidity than had men. In both the sexes, the relationship between age and the number of chronic diseases was almost identical, showing a weak association between age and the number of chronic diseases. The absence of significant differences by age-groups is contrasting to other studies performed in the West on the prevalence of multimorbidity (6,8,9) and is likely due to the fact that ageing of the population is rather a new phenomenon in Bangladesh. The proportion of people aged 60 years and above was 16.1% in the United States, 20.6% in the UK, and 22.4% in Sweden during 2000 whereas, in Bangladesh, it was only 4.9% in the same time period (4). Hence, ‘selective survival’ is likely to be a major explanation of the absence of age differences in our study. The concept of selective survival is that some people are more robust to fight against several illnesses and managed to be alive while others die. This selection might have taken place already at a younger age, by childhood illnesses, which may have produced cohorts more resistant to diseases at later age. The consequence of this reasoning is that reduction in infant and child mortality and morbidity will have a stimulating impact on the future prevalence of diseases at older ages. Still, the findings of the present study call for recognition of the urgent need of older persons, especially women, for healthcare services in Bangladesh.

The ‘non-poorest quintiles’ was associated with a higher prevalence of multimorbidity in this population. The persons who were relatively better-off suffered from more chronic conditions, e.g. arthritis and hypertension. It is important to stress ‘relatively better-off’, since we are dealing with a poor population. However, even within the poor communities, the differences do exist: unequal access to health services and differences in survival at infancy and early childhood. A stronger early biological selection in the poorest quintile (mortality of infant and children aged less than five years) may be part of the explanation of the lower prevalence of multimorbidity among the poorest segment of the study population. Further explorations, using a lifespan perspective, are probably needed to understand fully the higher prevalence of chronic diseases among the non-poorest quintiles.

We used the asset index rather than income to assess the socioeconomic status. Possession of assets and characteristics of infrastructure are easier to survey than income and are, therefore, less likely to be misreported. We purposively selected a sample from two blocks near the main health centre of icddr,b, blocks that are relatively more urbanized, resulting in a slight over-representation of the population in the non-poorest quintiles; hence, 17.5%, instead of 20%, of the study population were in the poorest quintile.

We found that, among the individual medical conditions, arthritis and hypertension were the most common conditions, similar to what has been reported in other studies (24). The prevalence of hypertension was also higher in the oldest age-group, which is consistent with the findings showing that increasing age is a risk factor for hypertension (23). The prevalence of hypertension was higher in the non-poorest quintiles but was only of borderline significance, which is consistent with findings of other studies that cardiovascular risk factors are associated with the economic development and where the mean level of population blood pressure is not correlated, or only weakly correlated, with economic factors (25). A study from the same research project reported a lower prevalence of arthritis using a rather stringent definition of the condition (26). The present study found a higher prevalence of arthritis by applying a revised definition based on a combination of medical history and current clinical examination. The prevalence of arthritis was higher among the illiterate people than among the literate people in this population. Higher prevalence rates were also observed among the people in the non-poorest group. The relationship among arthritis, literacy, and wealth needs to be further explored in this population.

Of the rural people participating in this study, women, illiterates, singles, and persons in the non-poor quintiles had the highest prevalence rates of multimorbidity. After adjusting for the sociodemographic characteristics, female sex and asset index were still associated with a higher risk of multimorbidity. Thus, the data suggest that our health system should focus on elderly women in particular. Other recent studies in Bangladesh have found a high prevalence of self-reported health problems among elderly persons, and approximately 80% of elderly women reported having four or more health problems (27). Gender might be linked to multimorbidity through various mechanisms. Vulnerability for co-occurring diseases may be due to genetic factors, including gender. Also the living and working environment, life-events, lifestyle, behavioural risk factors, or risk associated with socioeconomic status often differ between the genders and may affect the occurrence and outcome of multimorbidity. In our study, a random sample was selected from the community dwellers, and approximately 50% of the original sample participated in this study. People who initially participated in household interview but did not attend clinical examinations (n=152) were mostly women and elderly people (28). Thus, the high prevalence figures presented here may, in fact, represent an underestimation of the problem.

The high prevalence of multimorbidity observed in the study calls into question the very organization of our health services. Most developing countries, including Bangladesh, are least prepared to meet the challenges of societies with rapid increase in ageing population (29). The Revised Programme Implementation Plan (July 2003–June 2010) of the Health, Nutrition and Population Sector Programme of the Government of Bangladesh has only mentioned the possibility of developing a strategy for meeting the healthcare needs of senior citizens (30). The issue of the health of the elderly people rarely appears on the public-health agenda but findings from recent research showed that their health issues can be addressed through the existing primary healthcare infrastructure (31). The WHO has recently taken initiatives towards elderly-friendly primary healthcare and has developed ‘Age-Friendly Primary Health Care Centers Toolkit’ aiming at improving the primary healthcare responses to older persons, sensitizing and educating primary healthcare workers about the specific needs of their elderly clients, and providing guidance on how to make primary healthcare-management procedures more responsive to the needs of elderly people (32). The findings of our study showed that any effort to reorganize primary care for the elderly people should also consider the high prevalence of multimorbidity. Importantly, interventions that suit patients with a single disease may not be appropriate for patients with multiple conditions (33). The present study offers evidence to the present primary healthcare programmes in Bangladesh to concentrate on the elderly people with multimorbidity.

### Limitations

The study had several limitations. We could not include some diseases specifically important for the elderly people, such as diabetes and depression. Diabetes was not diagnosed in this population. Information on depression was not collected through clinical interview. In line with others, the chronic conditions with significant health impact were examined in the study. Regarding diagnoses of other health problems, e.g. respiratory tract infection, upper gastrointestinal tract disorders, lower gastrointestinal tract disorders, skin disease, and helminthiasis being mostly infectious/communicable, were not investigated in the study. Another aspect can be mentioned as the samples were from two nearby areas of the Matlab Hospital, the study people are relatively more urbanized.

### Conclusions

The study has shown that the prevalence of multimorbidity is high in a less-developed region, especially among women and in the non-poorest group of the aged population. Given the high prevalence and increased ageing of the population, clinicians and researchers should pay special attention to the diagnosis of multimorbidity among the elderly people. Since multimorbidity may cause significant cognitive and functional consequences, one of the relevant implications relating to our findings is that researchers and policy-makers should work together to develop effective intervention strategies and programmes to reduce the burden of multimorbidity. Furthermore, new healthcare models should be developed and evaluated to better meet the healthcare needs of elderly people with multimorbidity.

## ACKNOWLEDGEMENTS

The study was funded by the Department for International Development (DFID), UK, Swedish International Development Agency (Sida), and the Swedish Research Council. icddr,b acknowledges with gratitude the commitment of DFID, Sida, and Swedish Research Council to its research efforts. Dr. Masuma Akter Khanam was supported by the Swedish Institute. The authors express their gratitude to the participants and co-workers in the Poverty and Health in Ageing Project. The authors thank Dr. Atiqul Islam, Geriatrician, Karolinska Institutet hospital, for his valuable feedback on diagnosis of diseases.
